# No End in Sight; Assessing the Impact of Internet Gaming Disorder on Digital Eye Strain Symptoms and Academic Success

**DOI:** 10.3390/ejihpe14030035

**Published:** 2024-02-27

**Authors:** Georgios D. Floros, Mikes N. Glynatsis, Ioanna Mylona

**Affiliations:** 1 2nd Department of Psychiatry, Aristotle University of Thessaloniki, 54124 Thessaloniki, Greece; 2 Department of Ophthalmology, Hippokration General Hospital of Thessaloniki, 54642 Thessaloniki, Greece; mnglynatsis@gmail.com; 3 Department of Ophthalmology, General Hospital of Serres, 62100 Serres, Greece; milona_ioanna@windowslive.com

**Keywords:** Internet Gaming Disorder, Digital Eye Strain, academic achievement, diagnosis, predictive validity

## Abstract

Background: Internet Gaming Disorder (IGD) has been associated with symptoms of Digital Eye Strain (DES) and poor academic performance among adolescent students. The purpose of this study is to assess whether a student’s achievement of a specific academic goal within a short period of time can be directly predicted by symptoms of IGD and DES. Methods: This is a cross-sectional survey of 140 high school graduates who received an examination of visual acuity as a pre-requisite for entering the written admission examinations of law enforcement and military academies. The students completed the Digital Eye Strain Questionnaire (DESQ) and the Ten-Item Internet Gaming Disorder Test (IGDT-10) and stated their own evaluation of their chances for success. They were contacted following their admission examinations, and their success or failure to be admitted was recorded. Results: The students with IGD symptomatology were more likely to present with symptoms of DES. They were also more pessimistic about their chances of success in the subsequent written admission examinations; none succeeded, while the rest of the students recorded an expected rate of success. A combination of IGD and complaints related to the prolonged fixation of the upper body in a specific viewing position was the best predictor variable set for future success in admission examinations. Conclusions: IGD is associated with a failure to achieve academic success. Combining a factor for physical discomfort during prolonged sessions of gaming with the typical criteria for IGD may expand the predictive validity of the construct of gaming disorder.

## 1. Introduction

The concept of Internet Gaming Disorder (IGD) has been formulated over the last two decades as a concept to describe the emergence of addictive behavior related to online gaming [1]. While different studies have employed different terms for the same concept (e.g., ‘pathological/problematic online gaming’), the term IGD is more widely known and will be employed throughout this paper for consistency. Following the publication of a large number of epidemiological surveys, this concept attracted the attention of the American Psychiatric Association for possible inclusion in the new version of the psychiatric taxonomy. After considerable debate, the authors of DSM-V decided on the inclusion of ‘Internet Gaming Disorder’ in Section III as a condition warranting more clinical research and experience before it might be considered for inclusion as a formal disorder [2]. The focus on unique online behavior rather than on the full spectrum of possible online activities was warranted by the larger body of research data accumulated in particular for online gaming and its adverse outcomes, yet more research was deemed necessary for its formal inclusion in a future revision of the DSM-5. The concept has been well received by clinicians, yet several issues related to the validity of the proposed criteria remain [3]. In 2019, the World Health Organization included gaming disorder in the upcoming edition of the International Classification of Diseases (ICD-11), both in the predominantly online (designation 6C51.0) and predominantly offline form (designation 6C51.1) [4].

A loss of functioning due to IGD is the hallmark of the disorder, and at younger ages, functioning mainly refers to academic performance. A review of epidemiological studies up to May 2016 [5] found a number of studies pointing to lower school grades, skipped school classes, and truancy among IGD-affected adolescents, and more recent studies have confirmed those early findings; IGD has been recently associated with lower academic achievement in a study across seven European countries [6] and in a Lebanese high school student sample [7]. Adolescence is a sensitive age range, and a drop in academic functioning during this period could set the student up for failure to progress through classes and achieve hallmarks in later academic development.

Digital Eye Strain (DES) is a clinical syndrome which includes numerous eyesight symptoms, perceived as various degrees of irritation which interfere with further screen viewing, after lengthy engagement in front of a screen-enabled digital device [8]. The symptoms and signs which have been associated with DES are broadly divided into four categories:

1. Symptoms and signs of asthenopia (a subjective feeling of discomfort stemming from convergence insufficiency during prolonged screen time).
2. Symptoms and signs related to dry eye syndrome (inadequate lubrication of the eye due to a reduced presence of tears).
3. Symptoms and signs attributed to preexisting eye conditions (e.g., refractive disorders, presbyopia, amblyopic eye).
4. Symptoms and signs attributed to poor ergonomics (musculoskeletal problems are attributed to assuming body positions that generate tension for a prolonged period of time).

Our aim in this study was to assess the levels of IGD and DES in a random high school student population and assess whether they could be of significance in predicting academic success for an important academic goal.

Our research hypotheses were formulated as follows:

- Internet Gaming Disorder in our sample would be similar to corresponding levels in other surveyed countries, with males more likely to present with higher scores.
- Internet Gaming Disorder would be positively associated with measures of DES.
- Internet Gaming Disorder would be negatively associated with measures of student academic achievement.

## 2. Materials and Methods

### 2.1. Participants

All the participants were high school senior students in the province of Serres, Greece, who were candidates for admission examinations for law enforcement and armed forces academies. These candidates were required to pass an ophthalmic evaluation that assessed their visual acuity and level of correction (if any), with each academy setting its own prerequisite depending on the functional requirements. Typically, this precludes a number of pathological conditions, including amblyopia, color blindness, and high corrected ametropia (>6D), for all academies, while some academies set lower levels for corrected ametropia. The chances for success are relatively low due to high interest in entering these academies, with approximately one out of ten candidates succeeding in securing entry nationwide.

All entrants in the study were assessed in the Ophthalmology department of the General Hospital of Serres, Greece, during April–May 2023 as part of their obligatory ophthalmic evaluation. The students were invited, after passing their obligatory eyesight examination, to take part in this survey by completing an additional anonymous questionnaire. The students came from all different high schools in the region, and the sample was random with no particular order. Initially, two hundred consecutive candidates were approached for inclusion in the study. Forty-nine declined to participate, while eleven were unavailable for the follow-up questionnaire. The final sample consisted of 140 high school graduates, 82 male (58.6%) and 58 female (41.4%), with mean age = 17.61 years (SD = 0.853 years; range: 17–20 years).

### 2.2. Instruments

The Digital Eye Strain Questionnaire (DESQ) is a thirteen-item self-report scale in a yes-or-no format designed to offer a measure of complaints related to DES. The DESQ can be employed to reliably measure the symptomatology of DES in clinical populations who present either with eye issues or with excessive use of gaming and screen-enabled devices in general [9]. It has a three-factor structure, with the first factor relating to adaption issues following excessive use of screen-enabled devices, the second to dry eye issues, and the third to posture issues. Higher scores indicate more issues in terms of each individual factor and in an aggregated total score. No cut-off score has been proposed due to the heterogeneity of the three factors. Cronbach’s alpha for this study was 0.88, which was comparable to the validation study.

The Ten-Item Internet Gaming Disorder Test (IGDT-10) is a valid and reliable psychometric instrument used to assess IGD [10]. It consists of ten items that correspond to the nine proposed diagnostic criteria set forth by the DSM-5 for the preliminary diagnosis of IGD [2], with the last criterion broken down into two separate items that are assessed as a single one due to the complexity of its formulation. There are three possible responses to each item (‘never’, ‘sometimes’, and ‘often’), which are aggregated into a negative one (‘never’ and ‘sometimes’) scoring zero points and a positive one (‘often’) scoring one point. A total score of nine positive responses is nine points, with higher scores indicating higher severity and a nominal cut-off of 5/9 that corresponds to the proposed cut-off in DSM-5. Cronbach’s alpha for this study was 0.73, which was comparable to the validation study.

### 2.3. Procedure

This is a cohort study with a two-month follow-up on a single outcome. All participants completed an anonymous questionnaire that included their time spent daily using screen-enabled devices and online gaming (in hours) and their outlook for success on the coming entry exams (with four possible results, should pass/probably pass/probably fail/should fail the exams). DES symptomatology was assessed with the Digital Eye Strain Questionnaire, and IGD symptomatology was assessed with the Ten-Item Internet Gaming Disorder Test. The students then provided a means for contact, either by telephone or email/messaging service, and were contacted following the results of their entry examinations to confirm their outcome.

### 2.4. Data Analysis

According to our research hypotheses, the data analysis includes a comparison between the genders on their levels of Internet use, online activities, psychopathology indexes, and personality attributes. We carried out an ANOVA test of the differences in IAD scores and grade averages between the students, grouped by how helpful for their studies they perceived Internet use to be. Finally, two regression models were fitted for the prediction of study completion and psychopathology score in order to ascertain the best predictor variables. The “IBM SPSS Statistics 26” statistical package [11] was used for the data analysis.

## 3. Results

The mean time spent with screen-enabled devices was 7.15 h/daily (SD = 1.96 h; range: 3–12), and the mean time spent gaming was 3.19 h/daily (SD = 2.65 h; range: 0–10). Thirty-two students (22.9%) were classified as addicted to online gaming by the IGD-10 test, 26 of whom were male (81.3%) and 6 were female (18.8%). These two groups were compared with regards to their findings from the DES questionnaire. Table 1 presents the mean scores, standard deviations, Z values for the Mann–Whitney U test comparisons, *p* values, and effect sizes (d). All DES factors and the total score were statistically significantly higher in the addicted group. The effect sizes ranged from moderate for posture issues to high for the other variables.

A regression analysis, controlling for time spent online with any screen-enabled device, found that the IGD total score was a statistically significant predictor for the DES total score (*p* < 0.001), while time spent online was not (*p* = 0. 119; F(2;139) = 33.292; *p* < 0.001).

Table 2 presents the predictions made by the students regarding their possibility of success in the admission examinations, depending on the IGD grouping. The addicted students were more likely to believe that they would have an unfavorable outcome (chi-square (3) = 16.309; *p* = 0.001; effect size eta (η) = 0.276).

Table 3 presents the students’ success in the entry examinations, depending on the IGD grouping. The addicted students failed to succeed even once (chi-square (1) = 4.246; *p* = 0.039; effect size eta (η) = 0.174).

A binary logistic regression was conducted to assess the comparable strength of our demographic, general internet use, addictive online gaming, and DES variables in predicting whether or not a student would succeed in the entry examinations. The stepwise analysis concluded in two steps with the inclusion of the IGD total score and the posture issues DES factor, while gender, age, time spent online and in online gaming, and the other DES variables were not included in the model.

The two predictor variables considered together significantly predicted whether or not a student would succeed in the entry examinations (chi-square (2) = 29.173; *p* < 0.001). Table 4 presents the parameter estimates for the model, including odds ratios with 95% confidence intervals (lower and upper bounds). The final model has an estimated Nagelkerke R^2^ equal to 0.408, indicating that 40.8% of the variance in whether a student succeeded or not can be predicted from the linear combination of the variables in the model. This is a large effect, according to established guidelines [12], demonstrating the importance of those factors over the other psychosocial variables.

## 4. Discussion

This study employed a random high school student sample that had a stated goal of success in entry examinations that are hard to pass, with less than 10% of applicants gaining entry. Despite those arguably high expectations, there were a considerable number of students who, at the time of their visual function examination, presented with the addictive symptomatology of IGD. Their self-reported outlook of possible success at that time was bleak, and they all eventually failed to gain entry following their written examination a month down the line. All three initial hypotheses were confirmed with regards to the following:

- The incidence rates for IGD being higher for males than for females;
- The IGD total score correlating with all the DES factors;
- The students with IGD being unlikely to succeed in the admission examinations.

### 4.1. IGD Symptoms

While examining those results in detail, of note is the high incidence of IGD symptoms in this random student sample. This incidence does not necessarily directly translate to a diagnosis of IGD since there is a minimum timeframe of twelve months for symptom persistence as a prerequisite for a diagnosis according to both the DSM-V and ICD-11 classifications. However, compared to other recent studies on IGD that arrived at a pooled prevalence of 9.9% [13], the 22.9% percentage of students that crossed the diagnostic threshold appears high. The relative frequency of boys vs. girls is also considerably higher for boys, with roughly four boys affected for every girl. The difference in absolute numbers, however, could simply be attributed to incidence figures being typically higher than prevalence figures in any disorder where a large time frame of persisting symptomatology is required to assign a diagnosis. The larger-than-expected gender imbalance of 4.3:1 (the usual ratio is 2.5:1) may be attributable to peculiarities of the particular sample: a 2014 study of a Greek sample concluded with an even larger ratio of 6.2:1 [6]. Also, candidates for military and law enforcement academies may be more likely to identify with a warrior narrative that is common with many online games [14].

### 4.2. DES Symptoms

During the examination of visual function, none of the candidates who participated in the study had any preexisting eyesight problems that could contribute to DES symptoms. The findings indicated that the DES factors and total score were statistically significantly higher for the IGD-affected students, with moderate-to-high effect-size indexes denoting considerable clinical value for these findings. Online gaming, although recreational, is typically a competitive activity and requires a high level of attention and long periods of focusing onscreen without the appropriate timeouts that would reduce eye fatigue. There is a paucity of data on DES and online or video gaming in general; an Italian study of young children (mean age 6.9 ± 2 years) found that children who played video games for 30 min or more every day had more symptoms of asthenopia, an absence of fine stereopsis, and more refractive errors compared to children who did not [15]. A study in India that included 217 children with a mean age of 13 ± 2.45 years found that playing mobile games for longer than one hour per day was an independent risk factor for DES. A study of undergraduate medical students found that online gaming was one of the two activities with the highest prevalence of DES symptoms, the other being social media browsing. A Korean study of adult college students who played a video game without respite for four hours found that the subjects reported both physical and ocular discomfort as well as changes in binocular functions [16]. Of particular interest was the finding that neck and shoulder complaints were common due to the fact that these parts of the body remained fixed in the same posture while playing. There are two studies that have examined DES in online gaming addicts; a South Korean study followed a population of adolescents who exhibited risk for IGD for a full year and found an increase in the occurrence of dry eye symptoms [17]. The validation study for the DES questionnaire, which included a group of 150 ophthalmic patients and fifty addicted gamers, found that higher DESQ scores were associated with a higher total pathological internet use score, more time spent on a screen-enabled device, male gender, and belonging to the gaming-addicted group [9]. An unlikely source for data on DES and prolonged video gaming is the world of professional gaming, or ‘eSports’. In a survey of 65 eSports players, the most frequently reported complaint was eye fatigue, followed by neck and back pain [18]. These self-reported complaints would correspond to asthenopia and posture issues in DES. The time spent gaming by these professional players ranged from 5.5 to 10 h daily.

### 4.3. Predicting Students’ Academic Success

When examining DES and IGD in parallel regarding their predictive value in assessing the chances for success in the entry examinations, the DES posture issues factor and the IGD total score were the best combination of predictive factors.

The total score was a statistically significant predictor when controlling for total time spent with a screen-enabled device, demonstrating a qualitative difference in this particular online activity versus typical viewing activities with a digital screen. The students who were classified with a clinical level of IGD symptoms failed to achieve their stated goal of gaining entry into the academies they opted for, while the students who were not classified in the IGD group had a rate of success comparable to the expected nationwide average. The students in the IGD group were more likely to believe that they would not succeed, with only one stating that a positive outcome was a possibility, so apparently there was no lack of insight as to their level of preparedness. The eyesight evaluation is carried out close to the date of the written examination (no earlier than one and a half months before), so even if an individual does have the inclination to change his online gaming behavior, he/she is unlikely to have enough time for such an effort to have a meaningful effect on his/her academic performance. Also, an insight into the chances of success does not necessarily correlate with an insight as to the underlying cause, which is the accompanying disorder.

The finding of academic failure in IGD-affected students is in congruence with the relevant literature. A study of a large sample (N = 1928) of Norwegian adolescents [19] found video game addiction to be related to lower academic achievement; however, the time spent on video games alone did not correlate with lower achievement, pointing to the qualitative difference between addiction and high levels of engagement with online gaming. This finding was replicated in our student sample since the total time spent online was not a useful predictor of student achievement when controlling for IGD symptoms.

DES posture issues were the sole DES factor that was included in the analysis, despite being the DES variable with the smallest effect size in the individual between-group differences. It is possible that since symptoms of asthenopia and dry eye issues are more frequent in IGD, these symptoms do not have additional predictive value compared to the IGD total score, while posture issues capture an additional dimension related to muscle tension and extreme focus during gameplay. An IGD diagnosis assigns the same relative significance to all nine diagnostic criteria. This approach has been criticized as not clinically sound or statistically valid. Király et al. found the ‘preoccupation’ and ‘escape’ criteria to have very low discriminatory power during the process of creating the IGDT-10 questionnaire [10]. In their study using Item Response Theory (IRT), the researchers found that the ‘continuation’, ‘preoccupation’, ‘negative consequences’, and ‘escape’ criteria were associated with a lower severity of IGD, while the ‘tolerance’, ‘loss of control’, ‘giving up other activities’, and ‘deception’ criteria were associated with more severe levels of IGD. However, the DSM-5 threshold figure of five-ninths of the criteria for establishing a diagnosis was deemed appropriate. Ko et al. also found varying levels of diagnostic accuracy in the DSM-5 criteria while differentiating university students with IGD from remitted students, again validating the five-ninth threshold for diagnosis [20]. Interestingly, the DSM-5 set of criteria for IGD does not include any criterion related to obsessiveness or extreme engagement, an artifact from the differentiation of impulse control disorders (ICD) from disorders in the obsessive-compulsive spectrum (OCD) [21]. Supposedly, this type of gaming in the ICD-11 should relate to cases designated as ‘hazardous gaming’, yet the definition of this subtype of IGD is very vague and does not spell out obsessiveness and gaming for periods of continuous, uninterrupted sessions. Another missing aspect of addictive gaming in the DSM-5 criteria is the concept of ‘flow’ [22] felt during gaming. ‘Flow’ is defined as operating in a state that includes intense concentration, the merging of action and awareness, a loss of self-concern, control over one’s capabilities to engage in the activity, an altered sense of time, engagement with task goals, the receiving of immediate feedback, deep immersion in the activity, and an experience of intrinsic reward. Recent studies have found gamers participating more frequently and intensely in gaming to satisfy their craving for a state of flow [23,24].

Regardless of the etiology of excessively long-term gaming sessions, the set of IGD criteria currently employed does not directly assess their clinical significance. Assessing the musculoskeletal discomfort caused by these types of sessions may be an indirect way of filling that gap in the operational description of IGD. This assessment can be made indirectly with a self-report measure without complicating data gathering, as was the case in this study.

### 4.4. Limitations of the Study

This study did not address any other possible reasons for the poor academic performance of the IGD group. It is plausible that both poor academic performance and IGD symptoms are the end results of other stressors, social or psychological. It is also unclear whether addressing IGD symptoms at an earlier stage would have a positive impact on academic performance. The sole conclusion that can be supported by the data at hand is that the existence of IGD symptoms close to a crucial examination date is a negative predictor of success.

## 5. Conclusions

A random screening of visual function in senior high school students who were candidates for police and military academies found a high incidence of addictive online gaming symptoms. None of those students had either any history of or any current comorbid eyesight issues, yet those who demonstrated these symptoms were more likely to suffer from DES regardless of their total screen time. The students with IGD symptomatology were more pessimistic about their chances of success in the subsequent written entry exams, and rightfully so; none of them succeeded in the exams. On the contrary, the rest of the students recorded an expected rate of success. This study demonstrated that the implications of IGD symptoms could be detrimental to academic success in important educational goals, which may shape the future for a student. Thus, IGD symptomatology should be routinely screened for at the beginning of each school year with valid psychometric scales. Even if the actual IGD prevalence rates may be lower than what is suggested by the number of symptoms prevalent at any time, this high incidence of symptoms could point to detrimental effects when the desired goal is one with high competition among peers.

A possible field for future research would be an expansion of the timeframe, both before and after the admission examinations, for data gathering at multiple points; this would provide a clearer picture of IGD progression and assess its true prevalence in a relevant student sample since the symptomatology must be present for a period of twelve months before formally assigning a diagnosis. Nevertheless, even subclinical states may be detrimental to student success when a highly competitive event is imminent, such as an entry exam with a limited number of available slots. Being preoccupied with gaming could derail preparations for such an important event, even if the student is not clinically addicted; unfortunately, there is no relevant research as to the long-term impact of subclinical states of IGD in areas of functioning.

A combination of IGD and complaints related to the prolonged fixation of the upper body in a specific viewing position was the best predictor variable set for future success in the entry examinations. The latter complaints may fill in a gap in the current diagnostic set of criteria for IGD, which do not address the negative impact on the body and general discomfort from prolonged gaming. Future research should attempt to combine measures of psychological assessment with measures of ocular or body discomfort to improve diagnostic validity.

## Figures and Tables

**Table 1:** DES findings for the IGD groups with group comparisons.

	IGD Group	N	Mean	SD	Z	*p*	d
Adaption issues	Not addicted	108	3.24	1.11	5.4	<0.001	1.087
	Addicted	32	4.56	0.95			
Dry eye issues	Not addicted	108	2.50	1.17	5.56	<0.001	1.119
	Addicted	32	3.93	0.98			
Posture issues	Not addicted	108	0.94	0.76	2.33	0.02	0.469
	Addicted	32	1.31	0.86			
Total issues	Not addicted	108	6.69	1.57	7.67	<0.001	1.544
	Addicted	32	9.81	1.31			

**Table 2:** Predictions on future success in entry examinations.

			IGD Group		Total
			Not Addicted	Addicted	
Prediction	Should fail	Count	39	16	55
		% within IGD Group	36.1%	50.0%	39.3%
	Probably fail	Count	26	15	41
		% within IGD Group	24.1%	46.9%	29.3%
	Probably pass	Count	20	1	21
		% within IGD Group	18.5%	3.1%	15.0%
	Should pass	Count	23	0	23
		% within IGD Group	21.3%	0.0%	16.4%
Total		Count	108	32	140

**Table 3:** Student success by IGD group.

Success			IGD Group		Total	
			Not Addicted	Addicted		
No	Count		95	32	127	
	% within IGD Group		88.0%	100.0%	90.7%	
Yes	Count		13	0	13	
	% within IGD Group		12.0%	0.0%	9.3%	
Total	Count	108		32	140	

**Table 4:** Binary Logistic Regression Model of student success.

	B	S.E.	Wald Chi-Square (1)	*p*	Exp (B)	95% C.I. for EXP (B)	
						Lower	Upper
IGD total	−1.462	0.419	12.150	<0.001	0.232	0.102	0.527
DES posture issues	−1.191	0.508	5.499	0.019	0.304	0.112	0.822
Constant	2.535	1.076	5.547	0.019	12.618		

## Data Availability

Data are available upon reasonable request.
